# Assessment of binding interaction to salmon sperm DNA of two antiviral agents and ecofriendly nanoparticles: comprehensive spectroscopic study

**DOI:** 10.1186/s13065-023-00952-z

**Published:** 2023-04-19

**Authors:** Ahmed Faried Abdel Hakiem, Ahmed Mohsen Kamal El-Sagheir, Mohammed E. Draz, Niveen A. Mohamed, Ahmed Safwat Aboraia

**Affiliations:** 1 Pharmaceutical Analytical Chemistry Department, Faculty of Pharmacy, South Valley University, Qena, 83523 Egypt; 2 Medicinal Chemistry Department, Faculty of Pharmacy, Assiut University, Assiut, 71516 Egypt; 3 Department of Pharmaceutical Chemistry, Faculty of Pharmacy, Delta University for Science and Technology, Gamasa, 11152 Egypt; 4 Department of Pharmaceutical Chemistry, Unaizah College of Pharmacy, Qassim University, Unaizah, 5888 Saudi Arabia

**Keywords:** Daclatasvir and valacyclovir, Nitrogen doped orange quantum dots, An in-depth UV–visible spectroscopic investigation, The electrostatic and non-electrostatic kinetic parameters, Molecular modelling

## Abstract

The direct binding of antiviral agents; Daclatasvir and valacyclovir and green synthesized nanoparticles to salmon sperm DNA have been assessed in a comparative study. The nanoparticles were synthesized by the hydrothermal autoclave method and have been fully characterized. The interactive behavior and competitive binding of the analytes to DNA in addition to the thermodynamic properties were deeply investigated by the UV–visible spectroscopy. The binding constants were monitored in the physiological pH conditions to be 1.65 × 10^6^, 4.92 × 10^5^ and 3.12 × 10^5^ for daclatasvir,valacyclovir and quantum dots, respectively. The significant changes in the spectral features of all analytes have proven intercalative binding. The competitive study has confirmed that, daclatasvir, valacyclovir, and the quantum dots have exhibited groove binding. All analytes have shown good entropy and enthalpy values indicating stable interactions. The electrostatic and non-electrostatic kinetic parameters have been determined through studying the binding interactions at different concentrations of KCl solutions. A molecular modelling study has been applied to demonstrate the binding interactions and their mechanisms. The obtained results were complementary and afforded new eras for the therapeutic applications.

## Introduction

The DNA pair has four chemical bases; cytosine, guanine, adenine, and thiamine. Each base is attached to sugar and phosphate molecules giving a nucleotide. These nucleotides are arranged in a spiral manner into two strands what is known as the double helix resembling a ladder of phosphate and sugar molecules with rungs of the base pairs [1]. The binding to the DNA is considered the major goal of many drug molecules targeted to inhibit the cell activity. The conjugation with DNA could disturb the vital activities of the cell by modifying the transcription functions including essential proteins synthesis as well as gene expression, hence adversely affects replication. Molecules that behave in such manner could be deemed as efficient antimicrobial agents [2]. The binding of the small therapeutic molecules to DNA could be divided into number of classes based on their structural variation; Groove binding in the minor groove, alkylation by the chemical reaction with the DNA, cleavage of DNA chains, and the intercalation between base pairs [3]. Two modes are suggested to confirm the binding of small molecules to DNA; the irreversible covalent binding that is mainly achieved by alkylation cutting off whole DNA processes causing cellular damage [4]. The second mode is the reversible non-covalent binding, which could happen to a covalent one, it involves conjugation with DNA via groove binding, sandwiching (insertion), and intercalation through the phosphate clamp; separate coordination of phosphate moiety resulting in conformational and charge changes of the obtained conjugates [5]. Quantum dots are nanostructures of zero dimensions with electronic confinement in all directions. They provide improved intrinsic optical and redox characteristics with various surface passivation schemes introducing a new platform of natural light-activated antimicrobial agents. They have afforded many advantages including environment friendly, versatility for either to potency improved surface modification or synergistic adhesion to other antimicrobial agents, photostability, and considered excellent specific drug delivery vehicles [6, 7]. In addition, they could be synthesized from natural inexpensive sources with eco-friendly ways [8].

**Fig. 1:**
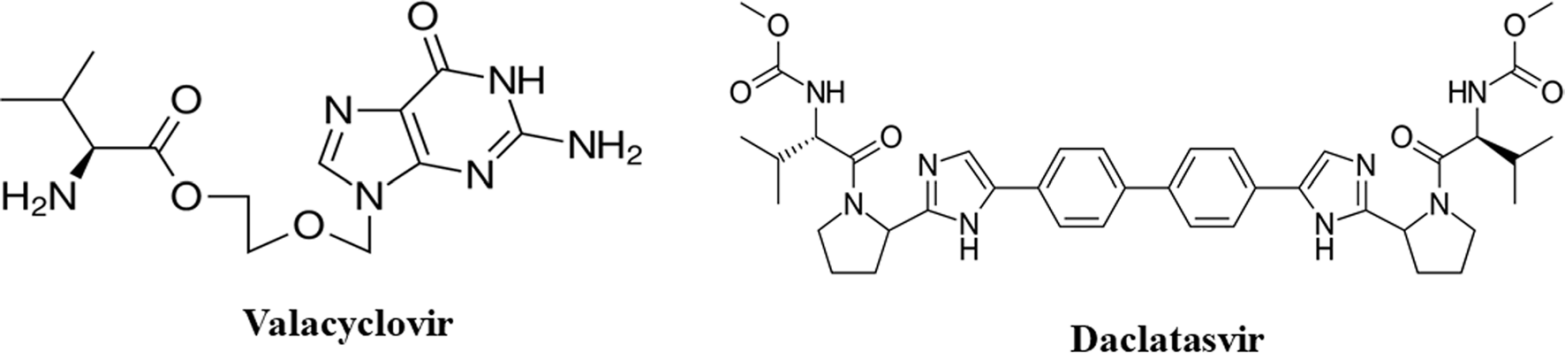
Chemical structures of the investigated molecules

Globally, the hepatocellular carcinoma caused by HCV and hepatitis B viruses infections is one of the major causes of death. Additionally, the chronic HCV infections cause cellular damage and liver cirrhosis [9]. Nowadays, researchers have shown much more interest to daclatasvir (DAC) molecule which is considered the game changer in different respects, chemically; methyl N-[(2S)-1-[(2S)-2-[5-[4-[4-[2-[(2S)-1-[(2S) -2- (methoxy carbonyl amino) -3- methyl butanoyl] pyrrolidine -2- yl] -1H- imidazol-5-yl] phenyl] phenyl]-1H- imidazol-2-yl] pyrrolidin-1-yl]-3-methyl-1-oxobutan-2-yl] carbamate, Fig. 1 [10]. The combination of DAC and sofosbuvir (SOF) has proven potential safety and efficacy in managing hepatitis C (HCV) infections as well as it enhances the stiffness of the liver and minimizes the portal hypertension and fibrosis [11]. DAC exhibits potential DNA destructive effect affording anticancer characteristics [12] thus it has dual mechanisms as antiviral and antitumor against the hepatocellular carcinoma. Recently, the DAC/SOF combination has been investigated in treatment of COVID19 patients and has shown potential efficacy in mild and moderate cases [13–15]. Valacyclovir (VAC) is an oral prodrug of acyclovir (its L-valyl ester), chemically; l-valine-2-[(2-amino-1,6-dihydro-6-oxo-9H-purin-9yl) methoxy] ethyl ester, Fig. 1. It exhibits potential antiviral activity against DNA viruses like herpesviruses and varicella zoster virus by inhibition of DNA polymerases terminating the chain elongation [16, 17]. There are current worldwide threats of viral pandemics and exceeding number of cancer patients as well as fears of future worse circumstances in theses respects. Accordingly, a great demand has evolved for finding new green alternatives like the orange QDs (OQDs) that suspected to be safe and hopefully acting more effectively than usual regimens. Additionally, precise evaluation of cellular toxicity and the antimicrobial activity of commonly prescribed c antiviral molecules like DAC and VAC could provide new eras in disease management. Few researches have studied DNA interactions with the investigated analytes, but their reports were restricted to detection of the conformational structural differences between the calf thymus DNA and the low molecular weight salmon sperm DNA (ssDNA). Many techniques were exploited in this respect like UV–visible spectrophotometry, fluorescence spectroscopy, isothermal coulometric titration and viscosity measurements [18]. An in-depth investigational study has been presented for the DNA binding interaction with VAC [19]. The DNA interaction behavior of DAC has been monitored throughout a comparative study with other antiviral agents based mainly on MALDI-TOF MS analysis and limited spectroscopic investigation for the spectral changes of only one concentration of both drug and DNA at three time intervals [12].

In this research work, a comparative and simple UV–visible spectroscopic study has been carried out for the interaction of ssDNA with the investigated analytes. The binding kinetics have been estimated, binding constant, binding mode through competitive interaction, effect of molar concentration, zeta potential measurements, and thermodynamic parameters. The selection of this spectroscopic technique as it provides a cost effective and accurate analytical tool as well as very weak luminescence criteria of VAC. Additionally, the tetrazine dye (TAR) has been also used as a determinant of the binding modes of OQDs, DAC and VAC through competitive displacement binding.

## Experimental

### Materials

Daclatasvir and TAR were kindly provided from GLOBAL NAPI Pharmaceuticals (Giza, Egypt), while VAC was gifted from EVA Pharma Pharmaceuticals (6th October city, Egypt). Salmon sperm DNA, Tris–HCl and ethylenediamine were purchased from Merck KGaA (Darmstadt, Germany). Potassium chloride crystals was purchased from the El-Nasr Company for Pharmaceutical Chemicals (Abozabaal, Egypt).

### Instrumentation

UV–Visible Spectrophotometer (PG Instruments Limited, T80, United Kingdom). Teflon-lined stainless steel hydrothermal autoclave (100.00 mL), locally designed and manufactured according to the standard dimensions. Mass spectrometer (Thermo Scientific, ISQ 7000 Single Quadrupole, Waltham, USA). X-ray diffraction pattern (Philips PW-1700) with wavelength Cu-Kα radiation at 40 kV and 30 mA settings, and at a diffraction angle range (20°–70°) with a step of 0.06°. High-resolution electron microscope (HRTEM) (JEM-100-CXII plus JEOL microscope working at 120 kV). Nicolet 6700 FTIR Advanced Gold Spectrometer, supported with OMNIC 8 software (Thermo Electron Scientific Instruments Corp., Madison, WI USA) for data processing. A 15 mm glass mortar and pestle and a hydraulic-press using a Perkin Elmer die press and Thermo-scientific (Fischer, USA) Qwik handi-press instrument were used to prepare the KBr OQDs sample discs.

### Synthesis of the nitrogen doped orange quantum dots

Fresh oranges were chopped and squeezed to get pulp-free juice. Modified procedure for a previous report [20] was utilized by mixing 55.00 mL orange juice with 0.50 mL of ethylenediamine in the autoclave and then allowed to be heated at 130 °C for 6 hrs. The autoclave was left to cool at room temperature. The dark brown solution was centrifuged twenty minutes at 5000 rpm. The supernatant was filtered through membrane filter (0.22 μm). The solution was then dialyzed with magnetic stirring for 24 h in one liter of deionized water. The water was replaced every 1 hr. Finally, the obtained solution was lyophilized to get the solid black nanoparticles of the nitrogen doped carbon QDs.

### Characterization of the OQDs

**Fig. 2:**
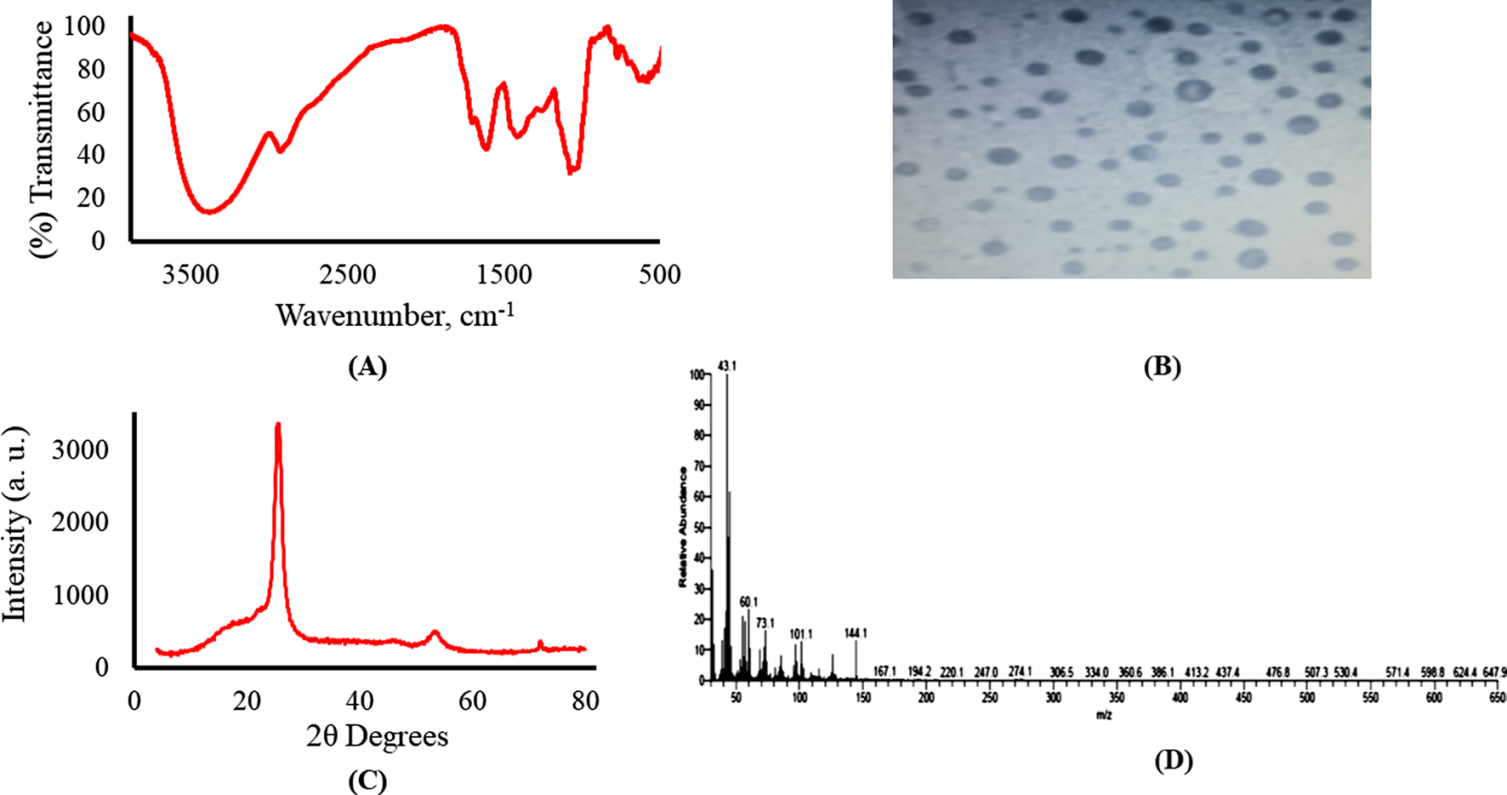
Characterization of the synthesized NQDs, **A** FTIR spectrum, **B** TEM image, **C** XRPD, **D** Mass spectrum

The fourier transform infrared spectrum (FTIR) has exhibited different vibrational features. A broad strong overlapping band extending from 3600 to 3050 cm^−1^ is attributed to O–H and N–H stretching vibrations. The medium broad band at 2930 cm^−1^ represents clearly a C-H stretching vibration. The medium shoulder at 1692 cm^−1^ has exhibited the carbonyl moieties confirming the formation of carboxylic acid moieties especially with the emerging broad band centered almost at 3300 cm^−1^. The formation of C═N stretching is indicated by the strong vibrational band at 1615 cm^−1^. The broad medium band and the weak shoulder at 1417 and 1250 cm^−1^ are assigned to the C═N and C-N stretching vibrations, respectively. The strong band at 1051 cm^−1^ is attributed to both C-OH and C-O-C stretching vibrations. The weak bands at 735 and 775 cm^−1^ refer to out-of-plane C-H bending [21, 22] [23], Fig. 2A. Further characterization has been applied by the transmission electron microscope (TEM). The resulting images have explored almost unisize nearly mono-spherical nanoparticles with size distribution ranged from 12.00 up to 20.00 nm, Fig. 2B. The X-ray powder diffraction (XRPD) pattern has revealed a sharp peak at 2θ = 25 (0.42 nm) and another weak one at 2θ of 53 (0.31 nm) indicating an amorphous carbon phase with a partial graphitic structure which is attributed to the abundance of amino, carboxylate and hydroxyl functional moieties, Fig. 2C. The mass spectrum, Fig. 2D of the synthesized NQDs has shown a peak at the end of the spectrum with molecular weight of 144.1 m/z which attributed to a stable OQDs fragment ion.

### Preparation of ssDNA solution

An appropriate amount of 0.02 g ssDNA was dissolved in 100 mL deionized water and stored away from light at 4 °C for one week. The stock ssDNA solution concentration was calculated to be 331.14 μM from the measured absorbance (at 260 nm), molar absorptivity (ε) of 6600 L.mol^−1^.cm^−1^ [24] and application of Beer-Lambert law. This stock solution was diluted with 0.01 M Tris–HCl solution to get working solutions of 2.50–40.00 μM. The purity was checked by determining the absorbance ratio at 260 nm/280 nm that was found to be more than 1.8 confirming that the ssDNA is almost free from protein [25].

### Preparation of working solutions

All studied analytes including the synthesized OQDs was found to have proper water solubility. Aqueous stock solutions of 0.20 mg/mL were prepared and were diluted furtherly with the same solvent to get 20.00 μg/mL working solutions of DAC, VAL and TAR as well as 100.00 μg/mL OQDs. The TAR solution has been diluted furtherly with 0.01 M Tris–HCl solution to get 12.50 μg/mL solution and then was mixed in equal volumes with 20.00 μM to get the working complex solution for the competitive study.

### Surface potentials of the studied analytes

**Fig. 3:**
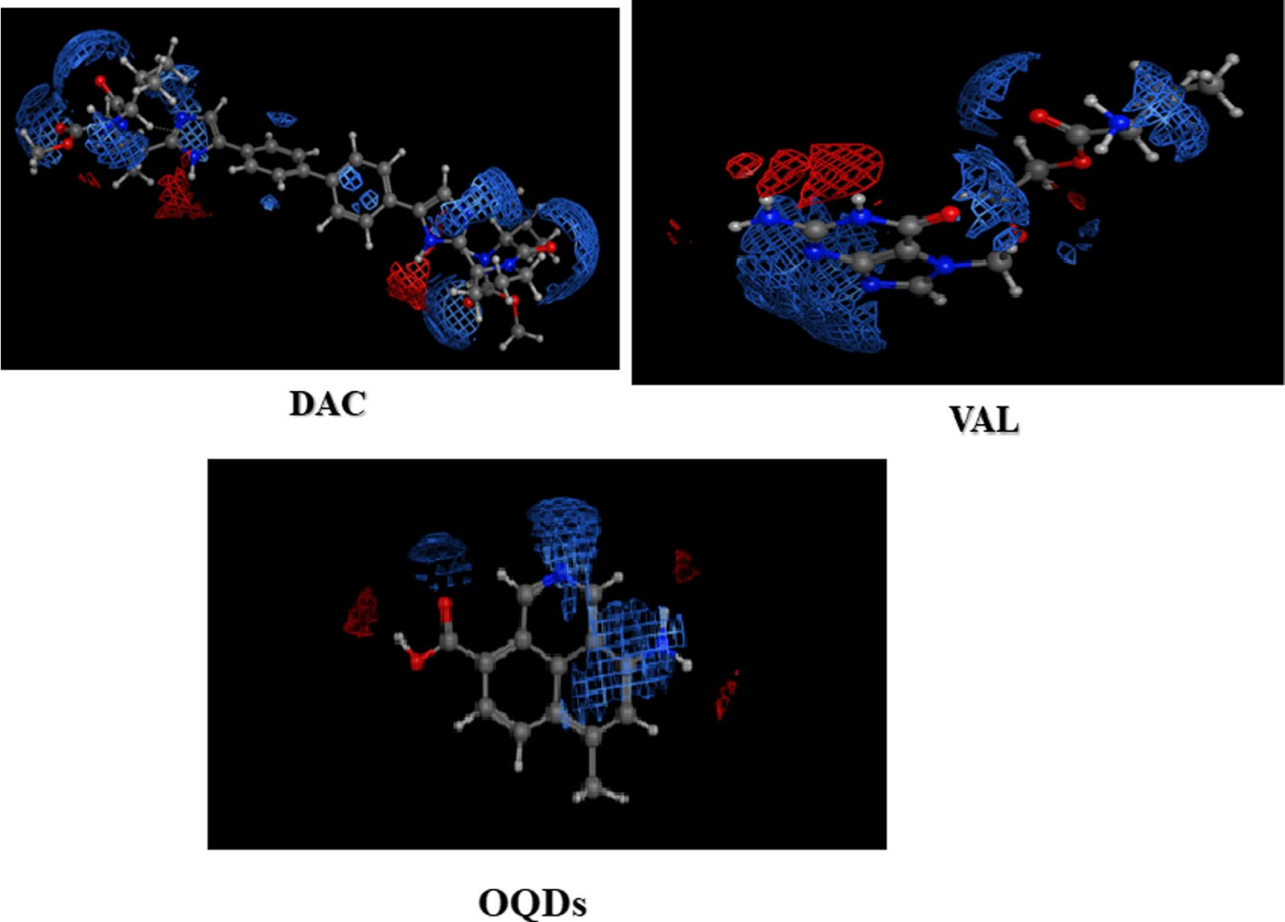
The electrostatic surface potentials of DAC, VAL and TAR, *where*, red and blue represent negative and positive charges, respectively

The electrostatic surface potentials were calculated for DAC, VAL and OQDs using the MOE 2020.01 software and they have shown abundance of positive charges, Fig. 3.

### Salt-concentration effect on the binding of the studied analytes and ssDNA

The lifetime of ligand (investigated drugs) on its binding sites at the biological host molecule (ssDNA) and hence its potency is a representation for the cumulative effects of both the non-electrostatic and electrostatic interactions. Changing concentration of either the ligand or the host provides a way to evaluate the role of non-electrostatic forces in the unbinding process as well as probing the change in the electrostatic ones [26] [27]. Assessment of the binding kinetics in varying electrolyte solution provides alternative and accurate way in this respect since biological molecules are amphiphilic in nature and their dissociation kinetics are greatly affected thermodynamically by the univalent salt concentration [28]. An inverse proportion governs the increasing in salt concentration and binding constants [29, 30]. This could be attributed to distance elongation between interacting charges and hence weakening the coulombic interaction (i.e. charge—charge interaction) and/or disturbance in the ionic distribution [31].

The interaction behavior of ssDNA with the studied analytes has been studied in KCl media of different concentrations from 0.00125 to 0.10 M since the more relevance of KCl to intracellular halophilic conditions and getting well defined bands with respect to NaCl [32]. The maximum studied KCl concentration was 0.10 M was because of that the binding constant doesn’t exhibit linear relationship with salt concentration above 0.125 M [33] and at 1.00 M or more the electrostatic interaction drops to zero and becomes mainly dependent on the non-electrostatic interaction. Hence, the stability of complex will be hindered as the electrostatic interactions will be interrupted and hence shows faster dissociation [34]. The concentration of salt [K^+^] has significant effect on binding interactions between the polyion (ssDNA monomer) and the counterion (studied analytes) since the ratio between their concentration above the unity (i.e. ratio between the least used experimental concentration of the added salt K^+^; 0.00125 M to that of ssDNA; 2.50 μM) [35].

### 3D Molecular docking

A molecular modelling and visualization study was carried out on synthetic double stranded DNA using Molecular Operating Environment software, MOE 2020.01. The structure of a parallel stranded DNA duplex at Atomic Resolution was obtained through the RCSB Protein Data Bank (PDB ID: 1JUU) [36]. The energy of docked ligand; DAC, VAL and NQDs (proposed fragments) were minimized with gradient RMSE of 0.0001 kcal/mol. The DNA structure was modelled by using the MOE QuickPrep protocol. The compounds were docked on double stranded DNA molecule using the method of Alpha triangle placement with Amber10: EHT forcefield. The Refinement was performed with Forcefield, and it was scored using the Affinity dG scoring system.

## Results and discussion

### Spectrophotometric assessment of the interaction ssDNA—studied analytes’ interaction

**Fig. 4:**
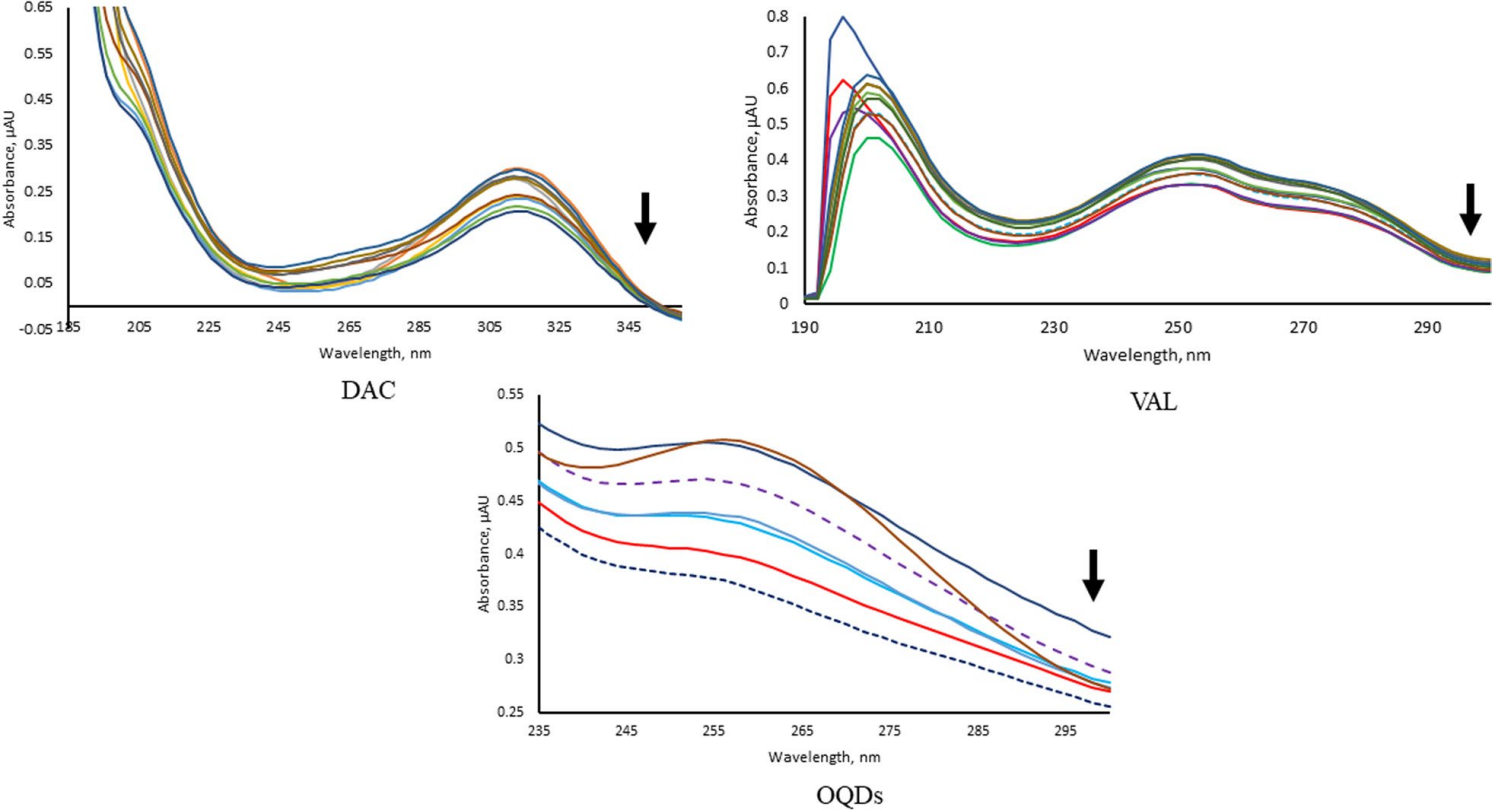
The UV spectral changes upon titration of the investigated analytes upon titration with increasing ssDNA concentrations of 2.50, 3.00, 5.00, 7.50, 10.00, 12.50, 15.00, 17.50, 20.00 and 22.50 µM

The studied analytes are considered excellent intercalators through vertical stacking to the backbone of the ssDNA without covalent bonding or hydrogen bonds breaking between bases pairs causing significant overlapping of p-electrons [37]. The analytes have exhibited different spectral behaviors upon titration of their fixed concentrations by increasing concentrations of ssDNA solution using 0.01 M Tris–HCl solution as blank, Fig. 4, Table 1. The large hypochromic effects with or without slight red shift confirm insertion of the planar aromatic rings of DAC and VAL in between the base pairs to different extents without disturbing the overall stacking pattern. Similarly, the OQDs have exhibited strong hypochromic effect as a result of stacking interaction between the ssDNA base pair and the heterocycle of OQDs (i.e. the FTIR characterization of the OQDs has shown bands at 1417 and 1250 cm^−1^ that are corresponding to the C═N and C-N stretching vibrations, respectively) [38]. These significant spectral changes have proved occurrence of large conformational changes expressed in decreasing helical twisting as well as length shortening [39]. Hence, their interactions with ssDNA are considered strong intercalation because of the strong hypochromic effects with almost insignificant bathochromic shifts varied from 2.00 to 6.00 nm [40–44]. It is worth noted that VAL has exhibited isosbestic points at 300 nm confirming its binding interaction. These spectral changes could be attributed to the decreasing in cube the distance between the analytes and ssDNA, hence minimizing the electronic interaction which is consistent with coupling between π^*^ electrons of the analytes and ssDNA leading to hypochromism which is encountered to a decrease in π–π* energy transition [45–48].

**Table 1:** The spectral effects of the added ssDNA on the maximum absorption spectra of the studied analytes

Analyte	Intensity	Wavelength	Δλ_max_
DAC	Hypochromic	314 nm	–
VAL	Hypochromic	196 nm	Bathochromic, 4 nm
	Hypochromic	252 nm	Bathochromic, 2 nm
OQDs	Hypochromic	252 nm	Bathochromic, 6 nm

### Competitive displacement assay

**Fig. 5:**
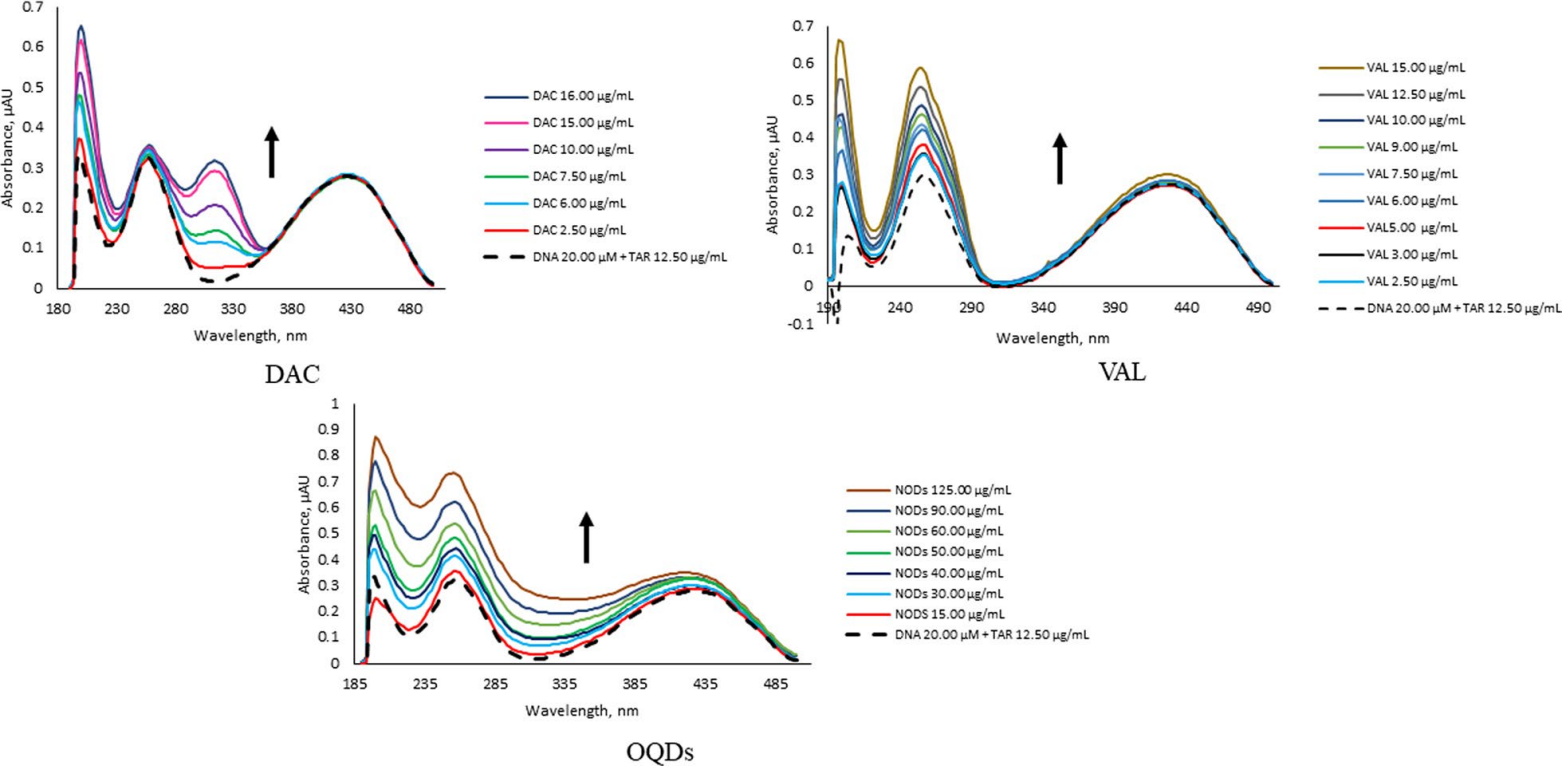
The competitive binding interaction study

The competitive replacement of a dye in conjugation with the DNA helix by a small molecule gives an indication that it has similar DNA binding fashion [49]. Tetrazine dye was selected for this study since it was evidenced to replace effectively the Hoechst 33258 dye that binds strongly to the minor groove of double-stranded B-DNA with specificity to AT-rich sequences [50]. The binding of ssDNA to the TAR dye was utilized efficiently to investigate the interaction modes between DAC, VAL and NQDs with ssDNA using 0.01 M Tris-HCl as blank. Hyperchromic spectral changes have taken place with the studied analytes as well as development of new absorption band at 316 nm with DAC due to the formation of a new complex. These spectral changes have indicated dynamic displacement of TAR dye by DAC, VAL and OQDs and hence confirming their minor groove binding to ssDNA [51], Fig. 5.

### The binding kinetic parameters at different electrolyte concentrations

The binding constants were calculated at different KCl concentrations. Linear relationships have been presented between the obtained binding constants versus the corresponding [K^+^] in order to obtain the total binding free energy change into its electrostatic and non-electrostatic contributions as well as the non-electrostatic binding constants in the light of the polyelectrolyte Eq. (1) [52, 53];

1 $$\mathrm{ln}\,{K}_{b}={\mathrm{ln}\,K}_{t}^{0}+Z{\zeta }^{-1}\left\{\mathrm{ln}\left(\gamma \pm \delta \right)\right\}+Z{\varvec{\psi}}(\mathrm{ln}[{M}^{+}])$$

**Fig. 6:**
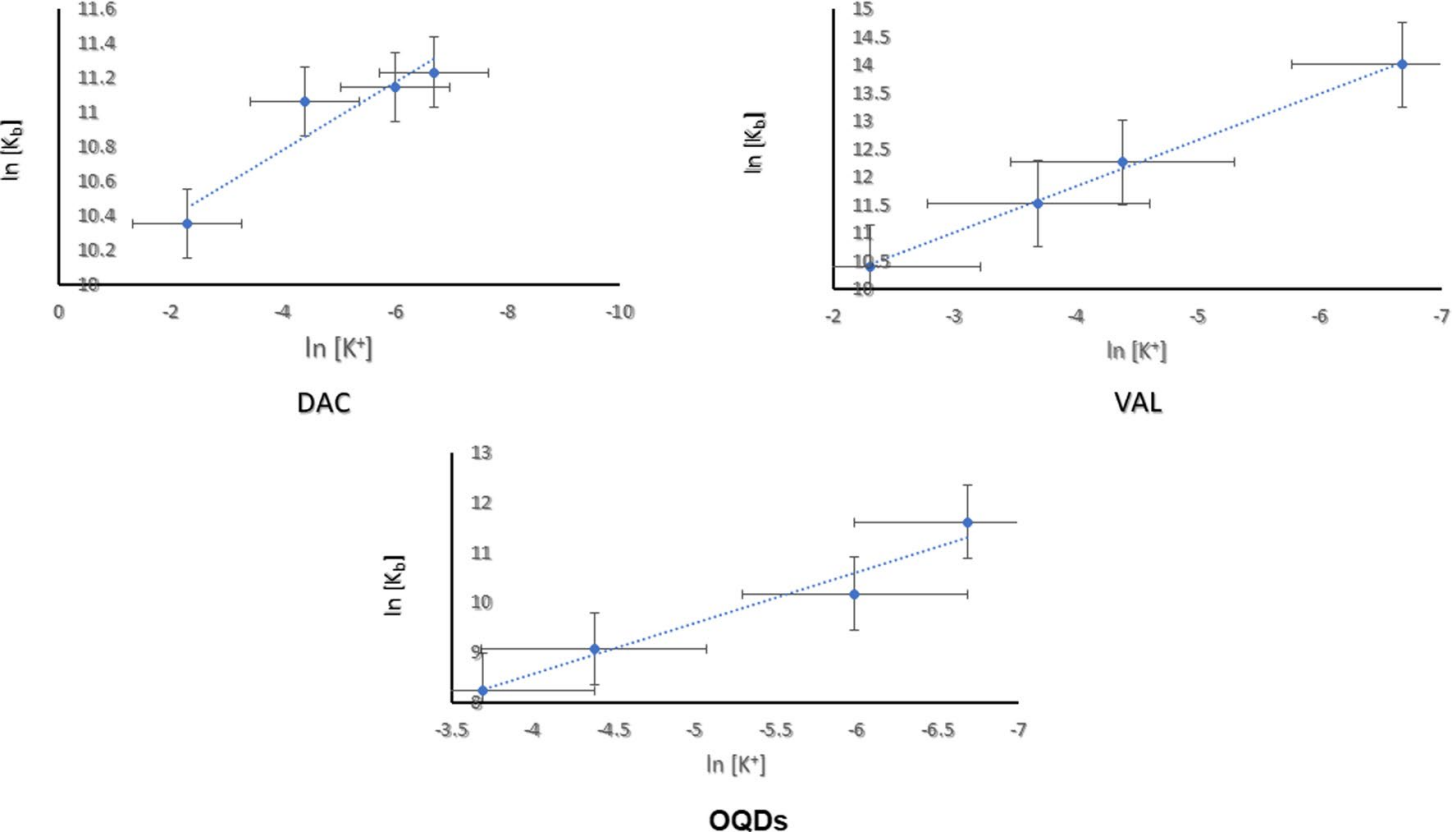
The effect of electrolyte concentration on the binding constants of the analytes to the ssDNA

*where*, *Z* is the partial charge on the binding ligand involved in the ssDNA phosphate groups interaction and could be estimated from the slope of the linear regression line between ln K_b_ and ln [K^+^], ψ is the number of cations associated with a phosphate group that are displaced on complex formation [54] and equals 0.88 for the B form of the double-stranded DNA and γ_±_ is the mean activity coefficient at different [K^+^], which was calculated in the light of Debye-Hiickel formula [55], Fig. 6.

The extent of association of monovalent counterions M^+^ with the polyions (ζ) and the fraction of the counterion condensed on an infinite polyion per structural polyion charge (δ) were found to be 4.2 and 0.76 for the B form of the double stranded DNA, respectively [56], the $K_{t}^{o}$ is the non-electrostatic contribution or stabilization to the overall binding constant K_b_ of the ssDNA—ligand complex and should be constant whatever the salt concentration. This term accounts for hydrophobic interactions; Van der Waal forces, translational, configurational and rotational entropies [57]. The studied analytes have shown decreased binding interaction by increasing the [K^+^]. In all cases, this could be attributed to favoring ion pairing and the associating release of of low molecular weight ions at low [K^+^]. Hence, the equilibrium will be shifted towards neutralization reactions and consequently complex formation [46].

On the other hand, they have exhibited almost the same or slightly increased non-electrostatic binding constants K^o^_t_ at higher potassium chloride concentrations in addition to increased contribution percentage in the total binding (% K_t_^o^/K_b_). This indicates that the electrostatic interaction is salt dependent and has minor effect on non-electrostatic forces. The binding interactions are energetically driven and possess the spontaneous character only the Gibbs free energy difference of the system between bound and free states (i.e. standard binding free energy, ΔG.^o^ and suggested to be at reference concentration of 1.00 mol/L) upon attaining equilibrium at definite pressure and temperature [58, 59]. It is related to the K_b_ and calculated through the this formula [60]

2 $$\Delta {\text{G}}^{o} = - {\text{RTln}}\,K_{b}$$

The electrostatic or polyelectrolyte (ΔG^o^_pe_) and the non-electrostatic contributions (ΔG^o^_t_) into the standard binding free energy are calculated getting the slope for the logarithmic plot between ln [K^+^] versus ln [K_b_] that represents the SK value and equals to the absolute value of Zψ (ψ = 0.88).

3 $$\Delta {\text{G}}_{pe}^{0} = {\text{(SK)RTln}}\,K_{b}$$

The non-electrolyte contributions (ΔG^o^_t_) is then obtained by the difference between the change in the standard binding free energy and the change in the electrostatic contribution [61].

4 $$\Delta {\text{G}}_{t}^{0} = \Delta {\text{G}}^{o} - \Delta {\text{G}}_{pe}^{0}$$

Since the light absorption is directly proportional to the square of the electric dipole transition moment. Hence, the changes in light absorption of polymers (i.e. DNA) is attributed to light induction in chromophores resulting in dipoles interactions [62] [63]. Hyperchromicity is considered one of the important evaluators for the strength of DNA binding interaction, it represents the effect of ligand binding on DNA molecule compaction as a result of electrostatic interaction [64]. The hyperchromicity percentage was calculated according to the following equation [65] [66]:

5 $${\text{Hyperchromicity }}\left( {{\% }} \right) = { }\varepsilon_{f } - { }\varepsilon_{b} /\varepsilon_{f } \times { 1}00$$

*where*, ε_f_, ε_b_ correspond to the extinction coefficients of the free and bound forms of the complex. All parameters were calculated for the studied analytes at different potassium chloride concentrations in accordance the above-mentioned equations and summarized in Table 2

**Table 2:** The kinetic parameters of the binding interactions between the studied analytes and the ssDNA at different [K^+^]

[K^+^]	K_b_ (M^−1^)	K_t_^o^	% H	ΔG^o^	ΔG^o^_pe_	ΔG^o^_t_	K_t_^o^/K_b_ (%)	ΔG^o^_t_/ΔG^o^ (%)
DAC								
0.1000	3.1253 × 10^4^	2.2671 × 10^4^	3.28	− 25.64	− 5.00	− 20.65	72.54	80.53
0.0125	6.3522 × 10^4^	2.2498 × 10^4^	4.37	− 27.40	− 5.34	− 22.10	35.42	80.65
0.0025	6.912 × 10^4^	2.2434 × 10^4^	10.93	− 27.61	− 5.38	− 22.23	32.45	80.52
0.00125	7.55 × 10^4^	2.2416 × 10^4^	11.67	− 27.83	− 5.42	− 22.41	29.70	80.52
VAL								
0.1000	3.2497 × 10^4^	5.958 × 10^3^	31.50	− 25.74	− 4.47	− 21.27	18.33	82.63
0.025	2.1224 × 10^5^	5.831 × 10^3^	12.55	− 28.55	− 4.96	− 23.40	2.74	81.96
0.0125	2.0634 × 10^5^	5.907 × 10^3^	20.01	− 30.40	− 5.27	− 25.11	2.86	82.60
0.00125	1.843 × 10^6^	5.811 × 10^3^	7.30	− 24.38	− 4.23	− 20.15	0.31	82.65
OQDs								
0.025	6.50 × 10^3^	98.74	12.30	− 21.75	− 0.50	− 22.24	1.52	102.25
0.0125	8.74 × 10^3^	97.61	41.70	− 22.48	− 0.51	− 22.99	1.12	102.27
0.0025	2.626 × 10^4^	95.95	5.83	− 25.21	− 0.57	− 25.78	0.36	102.26
0.00125	1.09858 × 10^5^	95.51	4.11	− 28.75	− 0.65	− 29.41	0.087	102.29

Close inspection of Table 2, it is evidenced that, all analytes have shown decreased binding constants by increasing [K^+^] and this confirms great contributions of electrostatic interactions into analytes—ssDNA conjugation. On the other hand, a slight increase that could be insignificant in the non-electrostatic forces, hence the binding interaction is mainly managed by the electrostatic ones. Accordingly, the percentage contributions of the non-electrostatic forces into the total binding K_t_^o^/K_b_ (%) has increased with increasing the electrolyte concentration. Daclatasvir provides the highest electronic environment among the analytes by two imidazole rings, biphenyl moiety, four carbonyl moieties, two acetyl moieties, and two pyrrolidine rings. The acyclovir molecule has less electronic configuration represented in purine nucleus with carbonyl moiety and substituted with amnio group. One imidazole ring, two phenyl and an azo moiety afford good electronic structure to the two molecules. Additionally, it was supposed that thermal treatment of OQDs has introduced many heterocyclic, aromatic, amino and surface carboxylic acid moieties. These structural chemical features have elucidated the descending order of the binding constants at physiological pH environment (provided by Tris–HCl) without electrolyte interference of 1.65 × 10^6^, 4.92 × 10^5^ and 3.12 × 10^5^ for DAC, VAL and OQDs, respectively. The lowest binding constant of TAR interprets changes in the absorption bands (hyperchromic effect) upon titration for its conjugate with the ssDNA with DAC, VAL and OQDs. Based on these experimental results, the electrostatic binding constant of DAC was logically and greatly hindered at different electrolyte concentrations. Both VAL and OQDs have almost similarly affected but lesser than DAC by the electrolyte concentration. The competition of K^+^ to the binding sites has caused varying spectral changes among analytes, it could decrease n–π* and π–π* transitions resulting in decreased % Hypochromicity with DAC or vice versa as observed with VAL and OQDs and hence confirming the binding interaction. The standard binding free energy, ΔG^o^ has shown inverse proportion with the electrolyte concentration and the negative signs refer to spontaneous exothermic interactions, affording reasonable behavior coincide with the numerical values of the binding constants at different electrolyte concentrations. Consequently, both the electrostatic and the non-electrostatic contributions into the binding free energy, ΔG^o^_pe_, ΔG^o^_t_, respectively, have been decreased at higher electrolyte concentrations. It is obviously noted that, the percentage contribution of the non-electrostatic binding free energy change into the total standard free energy hasn’t been changed for all analytes among all studied electrolyte concentrations. Both DAC and VAL have smaller percentages of nearly 80.00% than NQDs that has value around 102.00%. This indicates the higher electrostatic effect induced by the functional moieties’ rich structures of DAC and VAL. These almost constant percentages provide stability for ssDNA binding events of all studied analytes.

### Thermodynamic analysis

Parsing the binding free energy into its components represents an excellent approach to get detailed explanation about ssDNA interaction. The numerical values of standard enthalpy ∆H^o^, and standard enthalpy ∆S^o^ are considered the main determinants of the driving forces that monitor binding modes of the analytes with the ssDNA. The previous reports stated that when both ∆H^o^ and ∆S^o^ are less than zero, the interaction is dominated by hydrogen bonding or Van der Waals forces, while, if both are more than zero, the interaction is governed by hydrophobic forces but when ∆H^o^ ≤ zero and at the same time ∆S^o^ is more than zero, the electrostatic forces are considered the play maker in the binding interaction. These parameters are determined by construction of the Van’t Hoff plot in accordance to the following equation [67, 68]:

6 $$\ln K = \frac{{ - \Delta {\text{H}}^{o} }}{RT} + \frac{{\Delta {\text{S}}^{o} }}{R}$$

where, ln K represents binding constants obtained at four different investigated temperatures, R, is the gas constant and T, is the temperature in Kalvin.

**Fig. 7:**
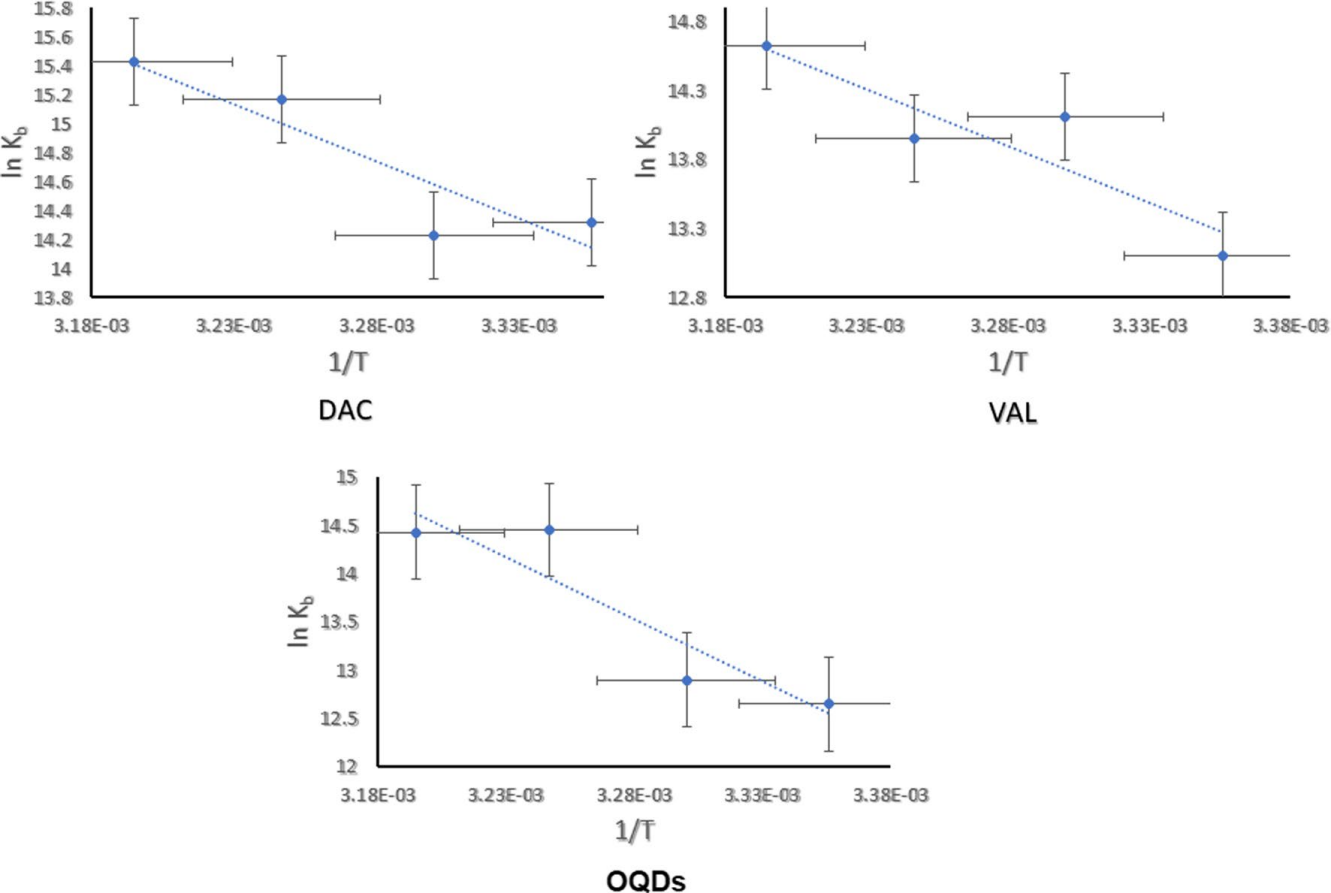
Thermodynamic study of the investigated analytes

The inverse proportions obtained among all analytes between ln K and 1/T, Fig. 7 as well the numerical values at Table 3 have evidenced that all bindings are governed by electrostatic interaction.

**Table 3:** Thermodynamic parameters of the studied analytes

K_b_ (M^−1^)	DAC	VAL	OQDs
K_b_ at 298	1.653125 × 10^6^	4.91966 × 10^5^	3.116 × 10^5^
K_b_ at 303	1.5074 × 10^6^	1.3418 × 10^6^	1.0082 × 10^6^
K_b_ at 308	3.85725 × 10^6^	1.1491 × 10^6^	1.8925 × 10^6^
K_b_ at 313	5.021667 × 10^6^	2.2474 × 10^6^	1.8509 × 10^6^
ΔH^o^	− 66.07	− 68.44	− 99.12
ΔS^o^	0.34	0.34	0.44

### Molecular modelling

**Fig. 8:**
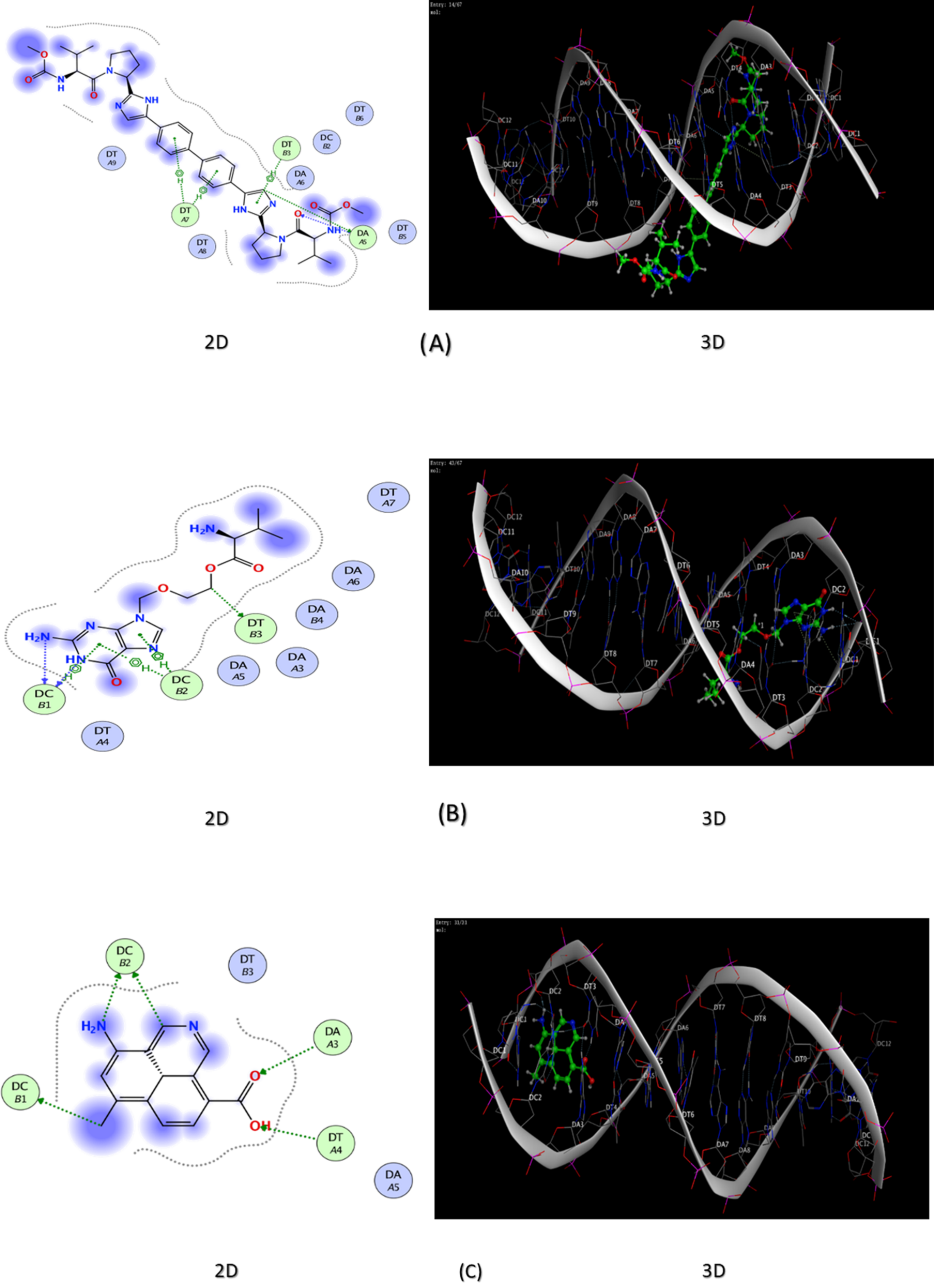
The molecular interaction between synthetic DNA and the investigated analytes; while, (**A**) corresponds to the 2D and 3D DAC-ssDNA interaction; (**B**) corresponds to the 2D and 3D VAL-ssDNA interaction; (**C**) corresponds to the 2D and 3D OQDs-ssDNA interaction

The docking study has provided a confirmatory insight into the analytes’ potential binding affinity to DNA molecule. The docking investigation showed the consistency with the results of the interaction kinetics. Daclatasvir has exhibited the highest exothermic interaction and binding affinity (the lowest binding score value) and hence the highest interaction spontaneity. On the other hand, NQDs (proposed fragments) was the least interacting molecule. All interactions with DNA bases mediated by H-bonding which formed mainly by heterocyclic aromatic moieties and all that drive the intercalation of the docked compounds into DNA molecule, Table 4, Fig. 8

**Table 4:** Energy scores (kcal/mol) and DNA interactions of DAC, VAL,and OQDs

Analyte	Energy score (S) (kcal/mol)	Interacting DNA bases
DAC	− 7.30	DT3, DA4, DT6, DA7, DT7, DT8
VAL	− 5.53	DA4, DT5, DA7, DT7, DT8
OQDs	− 5.19	DA3, DT4, DC1, DC2

*DA* DeoxyAdenine, *DT* DeoxyThymine, *DC* DeoxyCytosine

## Conclusion

This research has provided an intensive UV–visible monitoring of interaction behavior of the salmon sperm DNA with a number of small molecules and a green synthesized nanoparticles. Selection of analytes was based on the suspected anti-hebetic carcinoma activity of DAC besides its antiviral action, confirmation the potency of VAL as DNA antiviral agent and the suspected anticancer and antiviral activity of the ecofriendly synthesized nitrogen doped nanoparticles. Daclatasvir has exhibited the highest binding in the physiological condition, at the same time the green OQDs have shown promising binding. The interaction behavior of DAC, VAL and OQDs has been confirmed by efficient displacement of TAR from its complexes with ssDNA. The thermodynamic study confirmed the binding behavior through the acceptable numerical values of both entropy and enthalpy. Investigation of the binding kinetics in different KCl concentrations has proven that the electrostatic interaction is the main determinant of binding for all analytes. The 3D molecular docking study has introduced important complementary evidence about interactions and their mechanisms.

## Data Availability

The datasets used and/or analysed during the current study are available from the corresponding author on reasonable request.
